# Nuclear Pattern of CXCR4 Expression Is Associated with a Better Overall Survival in Patients with Gastric Cancer

**DOI:** 10.1155/2014/808012

**Published:** 2014-02-10

**Authors:** Bahram Nikkhoo, Ali Jalili, Shohreh Fakhari, Farshad Sheikhesmaili, Fardin Fathi, Daem Rooshani, Mohammad Ali Hoseinpour Feizi, Mehrnoush Nikzaban

**Affiliations:** ^1^Kurdistan Cellular & Molecular Research Center, Kurdistan University of Medical Sciences, Sanandaj, Iran; ^2^Kurdistan Liver & Digestive Research Center, Kurdistan University of Medical Sciences, Sanandaj, Iran; ^3^Department of Animal Biology, Faculty of Science, Tabriz University, Tabriz, Iran

## Abstract

*Introduction*. Previous studies have shown that stromal-derived factor-1 (CXCL12) and its receptor, CXCR4, play a crucial role in metastasis of various tumors. Similarly, it has been cleared that CXCR4 is expressed on the cell surface of gastric cancers. However, nuclear expression of CXCR4 and its clinical importance have not been yet studied. *Materials and Methods*. Herein, we studied the expression of CXCR4 in gastric samples from patients with gastric adenocarcinoma as well as human gastric carcinoma cell line, AGS, by employing RT-PCR, immunohistochemistry, and flow cytometry techniques. *Results*. RT-PCR data showed that CXCR4 is highly expressed on AGS cells. This was confirmed by IHC and FACS as CXCR4 was detected on cell membrane, in cytoplasm, and in nucleus of AGS cells. Moreover, we found that both cytoplasmic and nuclear CXCR4 are strongly expressed in primary gastric cancer and the cytoplasmic pattern of CXCR4 tends to be associated with a shorter overall survival than nuclear staining. In conclusion, we present evidence for the first time that both cytoplasmic and nuclear expression of CXCR4 are detectable in gastric cancer tissues. However, the role of both cytoplasmic and nuclear CXCR4 needs to be further elucidated.

## 1. Introduction

Gastric cancer remains as a major public health problem all over the world, and albeit its incidence and mortality have been gradually decreased, it is still the second cause of cancer related death [1]. The high mortality of gastric cancer is due to late diagnosis of the disease, indicating an urgent need for new diagnostic markers and treatment approaches [2].

Many studies have shown that metastasis of cancer cells from primary site resembles trafficking of normal cells which is governed by chemokines and their receptors, growth factors, adhesion molecules, and matrix metalloproteinases [3]. Solid tumors express chemokine receptors that provide them with guidance during migration to distant organs where their ligands are produced, showing a homing model similar to the one used by leukocytes to perform their immunological functions [4]. Consistently, accumulating evidence indicates that chemokines are involved in cell proliferation and chemoresistance of many tumors such as gastric cancer, breast cancer, and leukemia [5–7]. CXCR4 is a G-coupled receptor which binds its ligand, stromal-derived factor-1 (CXCL12), and plays a crucial role in retention of hematopoietic stem cells (HSCs) within the bone marrow [8]. Although CXCR4 is highly expressed in most of leukocytes and HSCs, CXCR4 expression is low or absent in most of normal solid tissues. In contrast, CXCR4 expression has been shown to be overexpressed in over 23 human cancers including breast, ovarian, melanoma, prostate, and gastric cancers [4].

The importance of CXCR4 axis in cancer is demonstrated by the fact that treatment of mice with CXCR4 antagonist leads to inhibition of metastasis of breast cancer [9]. Mounting evidence from various laboratories has indicated that malignant tumors, in contrast to benign tumors, express a high level of CXCR4 and the level of CXCR4 is correlated with metastasis to distant organs and reduced overall survival [10, 11]. Similar to many other cancers which express a high level of CXCR4, gastric cancers are also shown to express a high amount of CXCR4 which is correlated with tumor behaviors such as deep invasion to lymph nodes, liver metastasis, and poor differentiation [12, 13]. Moreover, overexpression of CXCR4 in gastric tumor has been demonstrated to be associated with development of malignant ascites and peritoneal carcinomatosis [13]. However, other reports indicated that CXCR4 expression was not associated with lymphatic invasion [14] and peritoneal metastasis [15]. Like other chemokine receptors, CXCR4 has been shown to be expressed on cell membrane and in cytoplasm of many tumors including gastric cancer [16], but not in the nucleus. However, recently published studies demonstrated that CXCR4 is expressed in nucleus of lung and colorectal cancers [17, 18]. Herein, we examined the expression of CXCR4 in gastric tissues from patients with gastric cancer and found that CXCR4 is expressed in cytoplasm and nucleus of most of the gastric primary tumors.

## 2. Material and Methods

### 2.1. Cell Culture

The AGS cell line (human gastric adenocarcinoma cell line) was purchased from Iran Pasteur Institute (Tehran, Iran) and was cultured in RPMI 1640 (Gibco, Manchester, UK) containing 10% FBS, at 37°C in a humid incubator with 5% CO_2_. Cells were subcultured when they reached approximately 80% confluence.

### 2.2. Reverse Transcription-Polymerase Chain Reaction

Expression level of mRNAs of CXCR4 and *β*-actin was evaluated in AGS cells. The reverse transcription-polymerase chain reaction (RT-PCR) procedures were carried out as previously reported [19]. Briefly, RNA was isolated using RNA extraction kit (Bioflux, Basel, Switzerland) and then transcribed into cDNA using Bioneer kit (Bioneer, Daejeon, South Korea). The RT-PCR was performed using primer sequences for human CXCR4 and *β*-actin (housekeeping gene) as listed in Table 1 and a thermocycler (Mastercycler, Eppendorf, Westbury, NY). Finally PCR products were electrophoresed on a 2% agarose gel containing ethidium bromide and gels were visualized under ultraviolet light using gel Documentation System (Bio-Rad, München, Germany).

### 2.3. Flow Cytometry Analysis

To determine surface CXCR4, AGS cells were stained as previously reported [19]. Cells were incubated with 10 *μ*g/mL of mouse anti-human CXCR4 (Clone 12G5, Santa Cruz, Heidelberg, Germany) or isotype antibody (Dako, Tehran, Iran) for 45 min on ice, then washed with FCM buffer (PBS containing 1% BSA), and incubated with goat anti-mouse FITC conjugated secondary antibody (Dako) for 30 min. Then cells were washed three times, fixed in 1% paraformaldehyde, and subjected to flow cytometric analysis (FACS Calibur, Beckman Dickinson, San Jose, CA). To detect intercellular CXCR4, surface CXCR4 was blocked by incubating the cells with 10 *μ*g/mL of mouse anti-human CXCR4 (Santa Cruz) for 45 min, and then cells were washed three times with FCM buffer. Cells were then permeabilized with 0.1% Triton X100 (Sigma) for 10 min, washed three times with cold PBS, and stained with either mouse anti-human CXCR4-PE/Cy5 or isotype antibody for 30 min at 4°C. Cells were next washed and ran by flow cytometry. Ultimately, CXCR4 expression was analyzed by FCS Express software (Los Angeles, CA).

### 2.4. Immunocytochemistry

In order to detect nuclear expression of CXCR4, AGS cells were cultured on cover slips for 24 h, washed three times, and fixed with 2% paraformaldehyde for 10 min at room temperature. To reduce nonspecific binding, cells were blocked with 2% bovine serum albumin and then incubated with 10 *μ*g/mL of mouse anti-human CXCR4 in a humidified chamber overnight. Next, cells were washed and stained with Ultra Tek HRP (ScyTek Laboratories, Utah) according to its manufacturer's instruction. Mayer's hematoxylin was used as counterstain.

### 2.5. Patients

Analysis of CXCR4 protein expression by immunohistochemistry was done on 43 patients who had undergone diagnostic endoscopy during 2004–2010 at the Gastroenterology Division of Tohid Hospital, Sanandaj, Iran. All specimens were histologically evaluated by conventional hematoxylin and eosin staining and intestinal type adenocarcinoma or diffused type diagnosis was confirmed for all of them. Patients enrolled in the study had not received any chemo- or radiotherapy before diagnosis. Patient's overall survival was considered as the duration between diagnosis and death (median survival: 327 days; range: 30–1800 days). Moreover, those patients who died of any disease other than gastric cancer were excluded from this study. A written informed consent was obtained from each patient.

### 2.6. Immunohistochemistry

Immunohistochemistry (IHC) staining was performed using the Ultra Tek HRP and anti-CXCR4 antibody according to the manufacturer's instruction. In brief, sections were prepared from gastric cancer blocks, mounted on charged slides with APES (Sigma), and fixed for 1-2 h at 60°C before staining. Then, the sections were deparaffinized in xylene and rehydrated in graded alcohol solutions. After antigen retrieval by heating (95°C) in citrate buffer (PH 6) for 15 min, endogenous peroxidase was blocked by treatment of sections with 3% hydrogen proxidase for 10 min. After blocking with 2% BSA for 10 min, slides were incubated with either anti-CXCR4 antibody (1 : 200) or mouse isotype antibody (1 : 100) diluted in antibody diluents (S3022; Dako) overnight in humid chamber at 4°C. Slides were washed and then incubated with the anti-mouse biotinylated secondary antibody for 20 min, washed three times, and then incubated with HRP-conjugated streptavidin for 20 min. Slides were washed and treated with 3,3′-diaminobenzidine (DAB) chromogen for 5 min and counterstained with Mayer's hematoxylin and mounted.

### 2.7. Evaluation of the IHC Staining for CXCR4

The slides were evaluated by our pathologist (B. Nikkhoo) who was blind to the patient's outcome and clinicopathological findings. The evaluation was carried out as previously reported [20]. The intensity was scored as low, moderate, and strong compared with background staining. The percentages of positive cells were estimated by calculating the ratio of the positively stained invasive tumor cells to the total invasive tumor cells. Nuclear versus cytoplasmic location of expression was also noted in each sample as previously reported [20].

### 2.8. Statistical Analysis

The SPSS package (SPSS, Inc., Chicago, IL) was used for statistical analysis. The association of CXCR4 expression with clinicopathological features was evaluated using Cox Regression Omnibus test. Survival rates were evaluated by applying Kaplan-Meier curves and *P* values <0.05 considered significant.

## 3. Results

### 3.1. CXCR4 Expression in Gastric Cancer Cell Line, AGS

**Figure 1:**
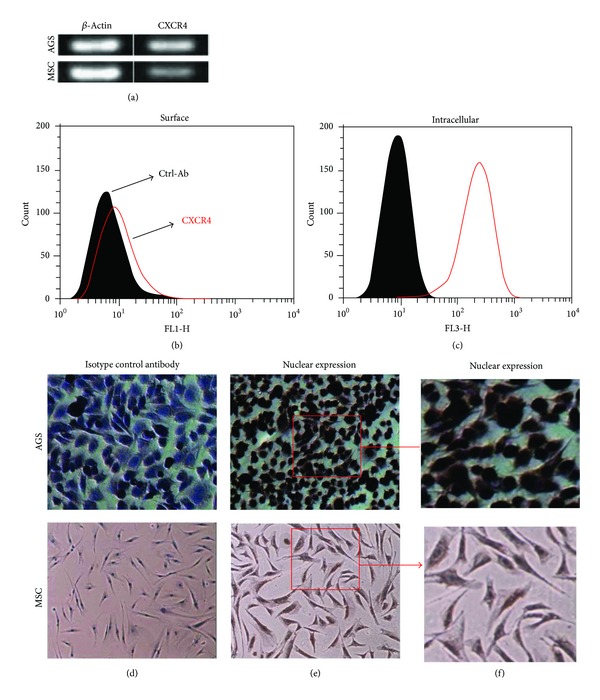
CXCR4 expression in gastric cancer cell line, AGS. Mesenchymal stem cells (MSC) and AGS cells were cultured to 80% confluence in 25 cm^2^, and total RNA was isolated. (a) CXCR4 transcripts levels were measured by RT-PCR. (b) Cell surface expression of CXCR4 was determined by flow cytometry. (c) Abundant CXCR4 level was detected in the cytoplasm of the AGS cells after permeabilization with Triton X100 and CXCR4 expression was detected by flow cytometry. (d-e) Immunocytochemical staining of AGS and MSC cells for CXCR4. Cells were cultured on cover slips for 24 h, fixed with 2% paraformaldehyde for 10 min, washed, and incubated with anti-CXCR4 overnight at 4°C. Then, cover slips were washed and stained with Ultra Tek HRP as stated in Material and Methods. (d) Isotype control antibody; (e) CXCR4 expression in nuclear and cytoplasm of AGS and MSC cells; (f) shows CXCR4 expression in the cells with a higher magnification.

First, we examined CXCR4 expression in gastric cancer cell line, AGS, by using RT-PCR and found that CXCR4 transcripts are highly expressed in this cell line (Figure 1(a)). Next, CXCR4 expression was determined by flow cytometry analysis. Our flow cytometry data indicate that CXCR4 is expressed at a low level on AGS cell surface. However, it is remarkably expressed in cytoplasm of this cell line (Figures 1(b) and 1(c)), indicating that cell surface expression of CXCR4 is not always correlated with CXCR4 transcripts or intracellular CXCR4, at least in AGS cell line. Moreover, we found that CXCR4 is also expressed in the nucleus of AGS cell line (Figures 1(e) and 1(f)).

### 3.2. IHC Staining Pattern for CXCR4 and Its Association with Clinicopathological Features in Gastric Cancer

**Figure 2:**
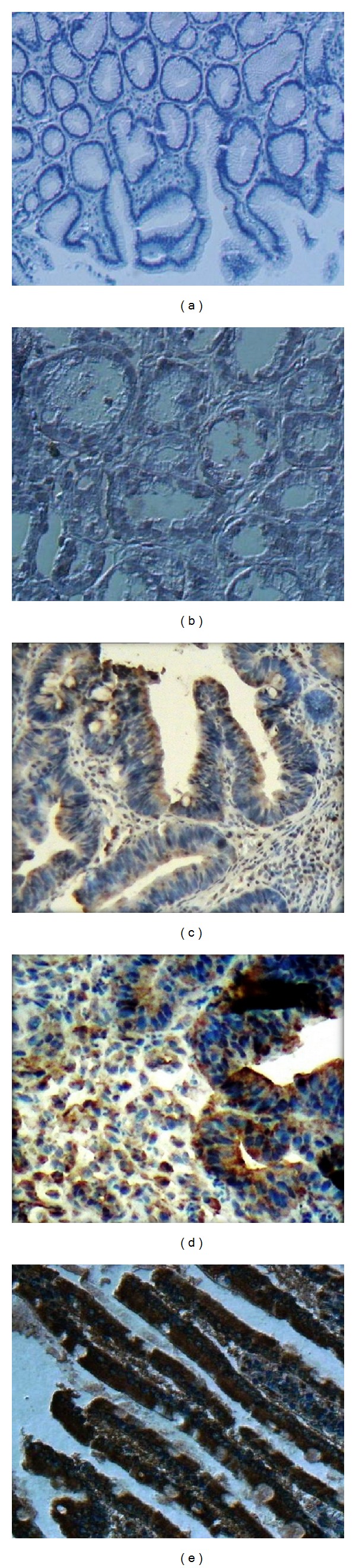
Representative photomicrographs of immunohistochemical staining for CXCR4 in primary gastric tumors. (a) No staining intensity for isotype control antibody. (b) Human atrophic gastritis samples were used as controls. (c) Weak staining of cytoplasmic pattern of CXCR4 in gastric cancer. (d) Moderate staining of cytoplasmic pattern of CXCR4 in gastric cancer. (e) Strong staining of cytoplasmic pattern with nuclear staining of CXCR4 in gastric cancer.

**Figure 3:**
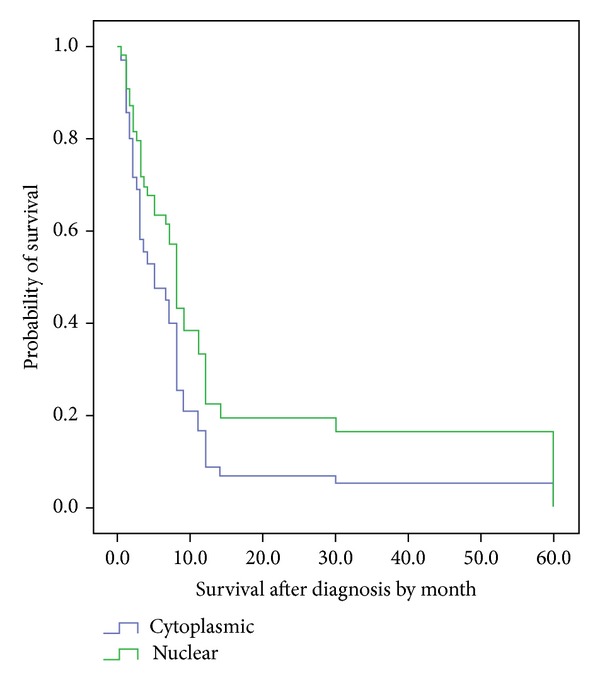
Correlation of overall survival of 43 gastric cancer patients with CXCR4 expression. Association of CXCR4 expression pattern with overall survival of the patients was evaluated by Cox Regression Omnibus test.

First, we used human atrophic gastritis samples as control for gastric cancer and stained them for CXCR4 expression and found that CXCR4 is expressed at a very low level in atrophic gastritis samples (Figure 2(b)). Then, samples from patients with gastric cancer were stained with CXCR4 expression and the pattern of CXCR4 expression was evaluated as stated in Table 2. Our data showed that predominant staining pattern of CXCR4 was cytoplasmic with a nuclear component defined as predominantly nuclear staining which occurs in 53.51% of samples. In contrast, cytoplasmic staining pattern of CXCR4 was found in 46.5% of samples (Figures 2(c)–2(e)). In addition to the staining of tumor cells, CXCR4 staining was observed in inflammatory cells and some parts of nonmalignant tissue adjacent to tumor cells. Next, to correlate the pattern of CXCR4 staining with clinical findings, we employed Cox Regression Omnibus test and found that patients with cytoplasmic CXCR4 showed a shorter overall survival than those with nuclear staining (Figure 3). However, this association did not reach statistical significance and a larger number of patients are required. In addition, we did not observe any other association between pattern of CXCR4 expression and other clinical findings such as age, gender, type of tumor, location, and histology (Table 3).

## 4. Discussion

It is well known that chemokines and their receptors exert many biological activities including cell migration and trafficking. Moreover, accumulating evidence indicates that tumor metastasis and trafficking of inflammatory cells is considered, at least partially, to be controlled by chemokines and their receptors [21]. In particular, CXCR4 expression is associated with more aggressive behavior of many tumors [22, 23]. Regarding gastric cancer, it has been previously shown that CXCR4 expression is not associated with lymph node metastasis of cancerous cells [14]. In contrast, a recent study has reported that CXCR4 is associated with lymph node metastasis in gastric cancer [24]. In the current study, we aimed to study CXCR4 expression in primary gastric cancers and attempted to correlate clinicopathological factors with the pattern of CXCR4 expression.

First, we examined CXCR4 expression on a well-known gastric cancer cell line, AGS, and found that, although CXCR4 expression is slightly low on the cells surface of AGS, its intracellular level is significantly high. In agreement with our data, another study previously reported that CXCR4 is expressed in cytoplasm of other gastric cancer cell lines [16]. We have recently found that AGS cells respond and migrate towards CXCL12 gradients (S. Fakhari, Advanced Biomedical Research, In Press) indicating that, even though the level of CXCR4 on the cell surface of AGS is slightly low, it is functional. In addition, we have recently examined the levels of CXCL12 by ELISA in the conditioned media of AGS cells and observed that these cells produce a high amount of CXCL12 (111 pg/mL ±33) (S. Fakhari, Advanced Biomedical Research, In Press), indicating that autocrine secretion of CXCL12 may result in endocytosis and ubiquitin-mediated degradation of CXCR4. This could explain the discrepancy between the high level of intracellular CXCR4 and low amount of cell membrane CXCR4 in AGS cells. Furthermore, our immunocytochemical data show for the first time that CXCR4 is also expressed in the nucleus of AGS cells. Collectively, our data demonstrate that CXCR4 is expressed on the cell membrane, in cytoplasm, and in nucleus of AGS cells. However, the biological function of nuclear CXCR4 has remained unknown.

It has been shown that CXCR4 is expressed in many tumor cells and those CXCR4-expressing tumors may show an organ-specific migration to the CXCL12-producing tissues/organs [22]. Although mounting data indicate that CXCR4 is expressed on the cell surface of gastric cancers [13], nuclear expression of CXCR4 in gastric tumors has not been yet reported. In the case of other cancers, CXCR4 has been shown to be expressed in both cytoplasm and nucleus of lung and colorectal cancers [17, 18]. Yoshitake et al. have shown that colorectal cancer patients with nuclear CXCR4 showed significantly more frequent lymph node metastasis than those with cytoplasmic CXCR4 [18]. By contrast, another study reported that nuclear CXCR4 was associated with a better outcome in patients with non-small-cell lung cancers [17]. These different results might be due to variations in methods, interpretation of the staining, and heterogeneous patient population. In the current study, we found that CXCR4 is expressed in cytoplasm of primary gastric cancer confirming the previous reports [13, 16, 24]. More importantly, we are demonstrating for the first time that CXCR4 is expressed in the nucleus of primary gastric cancer. When we attempted to correlate the pattern of CXCR4 staining with overall survival of the disease, we found that patients with nuclear CXCR4 staining tended to have a longer overall survival. We have to consider, however, that our study involved a limited number of patients (43 patients), and more studies with a larger number of gastric cancer patients are required to determine correlation of nuclear or cytoplasmic CXCR4 expression with survival of gastric patients.

In conclusion, we have found that CXCR4 is expressed in both cytoplasm and nucleus of gastric cancer cells and patients with nuclear CXCR4 expression have a better overall survival. However, extensive research is still required to clarify the biological function of nuclear CXCR4 in gastric cancers.

## Figures and Tables

**Table 1:** Primer sequences for RT-PCR.

Primer	Sequence	Product (bp)
CXCR4	Sense: ACAGTCAACCTCTACAGCAG	136
	Antisense: ATCCAGACGCCAACATAGAC	

*β*-Actin	Sense: AGATCATTGCTCCTCCTGAG	161
	Antisense: CTAAGTCATAGTCCGCCTAG	

**Table 2:** Consideration of positivity of CXCR4 expression.

Chemokine receptor	Pattern	Intensity	Percentage	Definition
CXCR4	Cytoplasmic	Moderate	>50%	Highly cytoplasmic expression
	Cytoplasmic	Strong	>30%	Highly cytoplasmic expression
	Nuclear	Weak, moderate, strong	>80	Predominantly nuclear expression

**Table 3:** Clinicopathological features of patients with gastric carcinoma according to pattern of CXCR4 expression.

	CXCR4 pattern expression		
	Cytoplasmic	Nuclear	*P* values
Age (years)	69.2 ± 11.55	68.56 ± 12.51	0.88

Gender			
Female/male	15/5	17/6	0.6

Type			
Intestinal	16	22	0.38
Diffuse	2	3	

Histology			
Differentiated	17	19	0.58
Undifferentiated	3	4	

Tumor location			
Upper	4	8	0.28
Middle	9	4	
Lower	8	5	
Whole	2	3	

## Abbreviations

CXCL12: Stromal-derived factor-1
HSCs: Hematopoietic stem cells
RT-PCR: reverse transcription-PCR.
